# Supplementary material for: An Examination of Multidimensional Time Perspective and Mental Health Outcomes
Source: Int J Environ Res Public Health. 2023 Mar 7;20(6):4688. doi: 10.3390/ijerph20064688 (PMC10048536; doi:10.3390/ijerph20064688)
Supplement: Supplementary file 1 [file ijerph-20-04688-s001.zip › Test-Retest Supplementary Material S2 FINAL.pdf]

Supplementary Material S2

Full Analyses of Covariance for Time Perspective (Orientation and Relation) and Mental Health Outcomes (Depressive Symptoms, Anxiety, and Rumination)

Variable	Depressive symptoms					Anxiety					Rumination				
	SS	*df*	*MS*	*F* ratio	η_p^2	*SS*	*df*	*MS*	*F* ratio	η_p^2	*SS*	*df*	*MS*	*F* ratio	η_p^2
Time orientation															
Covariates															
Age	1809.18	22	82.24	1.15	.17	342.10	21	16.29	0.81	.14	1154.77	25	46.19	1.12	.08
Gender	21.98	3	7.33	0.10	.00	147.41	3	49.14	2.45	.06	470.37	3	156.79	3.80*	.04
Anxiety	5962.49	21	283.93	3.96***	.40	N/A					N/A				
Depressive symptoms	N/A					2700.30	39	69.24	3.45***	.55	N/A				
Time orientation	419.16	5	83.83	1.17	.04	135.85	5	27.17	1.36	.06	739.60	6	123.27	2.99**	.06
Time relation															
Covariates															
Age	1774.25	22	80.65	1.14	.16	266.57	21	12.69	0.64	.11	1424.31	25	56.97	1.36	.10
Gender	81.76	3	27.25	0.38	.01	155.93	3	51.98	2.62	.07	560.10	3	186.70	4.45**	.04
Anxiety	7217.45	21	343.69	4.85***	.44	N/A					N/A				
Depressive symptoms	N/A					3034.93	39	77.82	3.93***	.58	N/A				
Time relation	382.72	3	127.57	1.80	.04	120.75	3	40.25	2.03	.05	407.79	3	135.93	3.24*	.03

Note. *SS* = sum of squares. *MS* = mean square. N/A = not applicable.

*p < .05. **p < .01. ***p < .001.
