# Supplementary material for: An Examination of Multidimensional Time Perspective and Mental Health Outcomes
Source: Int J Environ Res Public Health. 2023 Mar 7;20(6):4688. doi: 10.3390/ijerph20064688 (PMC10048536; doi:10.3390/ijerph20064688)
Supplement: Supplementary file 1 [file ijerph-20-04688-s001.zip › Test-Retest Supplementary Material S1 FINAL.pdf]

Supplementary Material S1

Full Multiple Linear Regression Models for Time Perspective (Feelings and Frequency) and Mental Health Outcomes (Depressive Symptoms, Anxiety, and Rumination)

Table S1

Positive Time Feelings and Mental Health Outcomes (Depressive Symptoms, Anxiety, and Rumination)

Variable	Depressive symptoms			Anxiety			Rumination		
	B	*SE B*	ß	*B*	*SE B*	ß	*B*	*SE B*	ß
				Past Positive					
Covariates									
Age	–0.10	0.13	–0.05	–0.14	0.07	–0.12	–0.23***	0.06	–0.19
Gender [a]	0.26	0.97	0.02	–0.59	0.54	–0.07	0.93*	0.46	0.11
Anxiety	1.04***	0.11	0.59	N/A			N/A		
Depressive symptoms	N/A			0.32***	0.03	0.56	N/A		
Past Positive	–0.29	0.82	–0.02	–1.05*	0.44	–0.14	–2.16***	0.44	–0.25
Constant	34.83***	4.85	N/A	3.77	3.04	N/A	33.78***	2.12	N/A
F ratio	24.84***			28.60***			13.97***		
R^2_{adj}	.35			.38			.10		
				Present Positive					
Covariates									
Age	–0.08	0.13	–0.04	–0.09	0.07	–0.07	–0.17**	0.06	–0.14
Gender [a]	0.33	0.97	0.02	–0.36	0.52	–0.04	1.07*	0.44	0.12
Anxiety	1.01***	0.12	0.57	N/A			N/A		
Depressive symptoms	N/A			0.29***	0.03	0.51	N/A		
Present Positive	–0.69	0.91	–0.05	–1.95***	0.47	–0.25	–3.14***	0.45	–0.35
Constant	36.04***	4.76	N/A	6.54*	2.91	N/A	36.28***	2.02	N/A
F ratio	25.02***			33.37***			22.83***		
R^2_{adj}	.35			.42			.16		
				Future Positive					
Covariates									
Age	–0.09	0.13	–0.04	–0.12	0.07	–0.10	–0.22**	0.06	–0.18
Gender [a]	0.19	0.98	0.01	–0.70	0.54	–0.08	0.93	0.47	0.10
Anxiety	1.02***	0.11	0.58	N/A			N/A		
Depressive symptoms	N/A			0.31***	0.03	0.55	N/A		
Future Positive	–0.66	0.82	–0.05	–1.07*	0.45	–0.14	–1.08*	0.47	–0.12
Constant	36.46***	5.01	N/A	4.21	3.16	N/A	31.08***	2.45	N/A
F ratio	25.05***			28.59***			7.50***		
R^2_{adj}	.35			.38			.05		

Note. An alpha adjustment was made to account for the six models that examined time feelings (shown in Tables S1 and S2; α < .008). N/A = not applicable.

[a] 1 = woman, 2 = man, 4 = trans man, and 5 = nonbinary/enby.

*p < .05. **p < .01. ***p < .001.

Table S2

Negative Time Feelings and Mental Health Outcomes (Depressive Symptoms, Anxiety, and Rumination)

Variable	Depressive symptoms			Anxiety			Rumination		
	B	*SE B*	ß	*B*	*SE B*	ß	*B*	*SE B*	ß
				Past Negative					
Covariates									
Age	–0.10	0.13	–0.05	–0.13	0.07	–0.11	–0.23***	0.06	–0.19
Gender [a]	0.27	0.97	0.02	–0.55	0.53	–0.06	0.88*	0.43	0.10
Anxiety	1.02***	0.11	0.58	N/A			N/A		
Depressive symptoms	N/A			0.31***	0.03	0.55	N/A		
Past Negative	0.54	0.78	0.04	1.14**	0.42	0.16	2.93***	0.36	0.40
Constant	32.39***	4.03	N/A	–3.01	2.60	N/A	18.21***	1.82	N/A
F ratio	24.98***			29.29***			28.60***		
R^2_{adj}	.35			.39			.20		
				Present Negative					
Covariates									
Age	–0.09	0.13	–0.04	–0.08	0.07	–0.06	–0.14*	0.06	–0.12
Gender [a]	0.28	0.97	0.02	–0.47	0.51	–0.05	0.99*	0.42	0.11
Anxiety	1.04***	0.12	0.59	N/A			N/A		
Depressive symptoms	N/A			0.29***	0.03	0.50	N/A		
Present Negative	0.16	0.87	0.01	2.15***	0.42	0.30	3.45***	0.39	0.43
Constant	33.25***	4.22	N/A	–5.90*	2.55	N/A	15.26***	1.95	N/A
F ratio	24.81***			36.64***			33.13***		
R^2_{adj}	.35			.45			.22		
				Future Negative					
Covariates									
Age	–0.09	0.13	–0.04	–0.10	0.07	–0.09	–0.19**	0.06	–0.16
Gender [a]	0.27	0.99	0.02	–0.83	0.53	–0.09	0.77	0.46	0.09
Anxiety	1.04***	0.11	0.59	N/A			N/A		
Depressive symptoms	N/A			0.31***	0.03	0.55	N/A		
Future Negative	0.03	0.91	0.00	1.57**	0.48	0.20	2.17***	0.46	0.25
Constant	33.59***	3.94	N/A	–3.28	2.55	N/A	21.57***	1.92	N/A
F ratio	24.79***			30.61***			13.40***		
R^2_{adj}	.35			.40			.10		

Note. An alpha adjustment was made to account for the six models that examined time feelings (shown in Tables S1 and S2; α < .008). N/A = not applicable.

[a] 1 = woman, 2 = man, 4 = trans man, and 5 = nonbinary/enby.

**p* < .05. ***p* < .01. ****p* < .001.

Table S3

Time Frequency and Mental Health Outcomes (Depressive Symptoms, Anxiety, and Rumination)

Variable	Depressive symptoms			Anxiety			Rumination		
	B	*SE B*	ß	*B*	*SE B*	ß	*B*	*SE B*	ß
				Past frequency					
Covariates									
Age	–0.08	0.13	–0.04	–0.12	0.07	–0.10	–0.13*	0.06	–0.11
Gender [a]	0.36	0.93	0.02	–0.52	0.54	–0.06	1.00*	0.43	0.11
Anxiety	0.88***	0.11	0.50	N/A			N/A		
Depressive symptoms	N/A			0.30***	0.04	0.52	N/A		
Past frequency	3.15***	0.82	0.24	1.06*	0.49	0.14	3.50***	0.42	0.42
Constant	23.74***	4.31	N/A	–2.97	2.69	N/A	11.22***	2.37	N/A
F ratio	30.60***			28.24***			30.18***		
R^2_{adj}	.40			.38			.21		
				Present frequency					
Covariates									
Age	–0.10	0.13	–0.05	–0.12	0.07	–0.10	–0.23***	0.06	–0.19
Gender [a]	0.28	0.97	0.02	–0.58	0.54	–0.06	1.04*	0.47	0.12
Anxiety	1.05***	0.11	0.60	N/A			N/A		
Depressive symptoms	N/A			0.33***	0.03	0.58	N/A		
Present frequency	0.43	0.89	0.03	–0.54	0.50	–0.07	–0.47	0.44	–0.06
Constant	32.04***	4.90	N/A	1.45	3.07	N/A	28.75***	2.42	N/A
F ratio	24.89***			26.82***			6.07***		
R^2_{adj}	.35			.37			.04		
				Future frequency					
Covariates									
Age	–0.10	0.13	–0.05	–0.12	0.07	–0.10	–0.21**	0.07	–0.17
Gender [a]	0.26	0.97	0.02	–0.54	0.55	–0.06	1.09*	0.47	0.12
Anxiety	1.05***	0.11	0.60	N/A			N/A		
Depressive symptoms	N/A			0.33***	0.03	0.58	N/A		
Future frequency	–0.13	0.75	–0.01	0.29	0.42	0.04	0.81	0.43	0.10
Constant	34.27***	5.07	N/A	–1.92	3.20	N/A	22.82***	2.65	N/A
F ratio	24.80***			26.53***			6.93***		
R^2_{adj}	.35			.37			.05		

Note. An alpha adjustment was made to account for the three models that examined time frequency ($\alpha < .017$). N/A = not applicable.

[a] 1 = woman, 2 = man, 4 = trans man, and 5 = nonbinary/enby.

**p* < .05. ***p* < .01. ****p* < .001.
